# Within-species divergence in the seminal fluid proteome and its effect on male and female reproduction in a beetle

**DOI:** 10.1186/s12862-015-0547-2

**Published:** 2015-12-02

**Authors:** Julieta Goenaga, Takashi Yamane, Johanna Rönn, Göran Arnqvist

**Affiliations:** Animal Ecology, Department of Ecology and Genetics, Evolutionary Biology Centre, Uppsala University, Norbyvägen 18D, SE-752 36 Uppsala, Sweden; Aarhus Institute of Advanced Studies, Aarhus University, Høegh-Guldbergs Gade 6B, 11 8000 Aarhus C, Denmark

**Keywords:** Acps, Accessory glands, Proteomics, Speciation, Sperm competition, Sexual conflict, Bruchidae

## Abstract

**Background:**

Male seminal fluid proteins (SFPs), transferred to females during mating, are important reproductive proteins that have multifarious effects on female reproductive physiology and that often show remarkably rapid and divergent evolution. Inferences regarding natural selection on SFPs are based primarily on interspecific comparative studies, and our understanding of natural within-species variation in SFPs and whether this relates to reproductive phenotypes is very limited. Here, we introduce an empirical strategy to study intraspecific variation in and selection upon the seminal fluid proteome. We then apply this in a study of 15 distinct populations of the seed beetle *Callosobruchus maculatus*.

**Results:**

Phenotypic assays of these populations showed significant differences in reproductive phenotypes (male success in sperm competition and male ability to stimulate female fecundity). A quantitative proteomic study of replicated samples of male accessory glands revealed a large number of potential SFPs, of which ≥127 were found to be transferred to females at mating. Moreover, population divergence in relative SFP abundance across populations was large and remarkably multidimensional. Most importantly, variation in male SFP abundance across populations was associated with male sperm competition success and male ability to stimulate female egg production.

**Conclusions:**

Our study provides the first direct evidence for postmating sexual selection on standing intraspecific variation in SFP abundance and the pattern of divergence across populations in the seminal fluid proteome match the pattern predicted by the postmating sexual selection paradigm for SFP evolution. Our findings provide novel support for the hypothesis that sexual selection on SFPs is an important engine of incipient speciation.

**Electronic supplementary material:**

The online version of this article (doi:10.1186/s12862-015-0547-2) contains supplementary material, which is available to authorized users.

## Background

In virtually all animals with internal fertilization, male accessory reproductive glands produce a complex cocktail of seminal fluid proteins (hence, SFPs) that are transferred to females during mating. These reproductive proteins have attracted much research interest during the last two decades for at least four related reasons. First, they exhibit an extraordinary and apparently redundant diversity. For example, *Drosophila melanogaster* males transfer well over 100 SFPs to females [1] and the seminal fluid of humans contains over 900 proteins [2]. Second, in model taxa, experimental obstruction of SFPs are known to affect a range of important reproductive phenotypes, such as sperm survival, male fertility, sperm usage by females, female ovulation/egg production, male competitive fertilization success and female life span and receptivity to remating [3–5]. Third, some of the genes encoding SFPs evolve very rapidly, to the point where they are some of the most rapidly evolving genes known [6–8], and a sizeable proportion of these genes show signs of positive Darwinian selection [9–14]. Finally, because SFPs are ubiquitous, affect reproductive success and often evolve divergently, genes encoding SFPs are key candidate speciation genes in several taxonomic groups [6, 15, 16].

An understanding of the divergent evolution of SFPs requires an understanding of the mechanism of selection that acts upon SFP variation. Here, the primary paradigm states that strong postmating sexual selection among males generates evolution of SFPs and that male-female coevolution then sparks divergent evolution in both SFPs and the female molecules that interact with SFPs [6, 17–19]. This tenet has received comparative support, for example from correlations across species between the inferred degree of sperm competition and the rate of evolution of SFPs in primates [11, 20], rodents [21, 22] and insects [23].

A large body of detailed experimental work, involving for example removal or ectopic expression of single SFPs using mutants or RNAi, has unveiled the function of key SFPs in model taxa [4]. However, virtually all of what we know about SFP evolution is based on comparisons between species [19]. Although the interspecific approach has been very illuminating, it also suffers from limitations. For example, the fact that the seminal fluid is such a multivariate phenotype/genotype makes it difficult to evaluate patterns of covariation, or indeed the lack thereof, between specific SFPs and species characteristics [21]. Moreover, the strength and nature of selection for a given SFP can differ among species, given that these proteins may have different effects in different species. Finally, interspecific approaches generally do not allow functional assays, where the reproductive effects of particular SFPs are assessed experimentally, simply because species are more or less reproductively isolated. For these reasons, studies focusing on within-species variation would allow us to test for selection and to characterize divergence, which is necessary to unveil the processes of SFP evolution [6, 19, 24–26].

The sexual selection paradigm of SFP evolution makes at least three critical intraspecific predictions. First, reproductive traits under sexual selection are generally among the most rapidly and divergently evolving traits during incipient speciation [27]. This predicts that seminal fluid composition should differ markedly between allopatric populations. Second, because the seminal fluid is such a multivariate phenotype, haphazardous mutation-order events [28] should render sexual selection and male-female coevolution to steer populations along different multivariate coevolutionary trajectories [17]. This predicts that diversification among populations will, itself, be multidimensional. Third, because sexual selection acts on traits related to reproductive success, we predict interpopulation divergence in SFP abundance to be functional in the sense that it affects important reproductive phenotypes. This important prediction is unique to the sexual selection paradigm. Unfortunately, there are very few within-species in-depth studies of SFP variation. Within *Drosophila*, several SFP genes are known to exhibit high rates of divergence across populations and at least some show significant molecular indices of directional selection [9, 24]. Moreover, studies of standing sequence variation in protein coding SFP genes in *D. melanogaster* have successfully associated allelic variation in a few candidate SFP genes with important reproductive phenotypes [29–31]. However, we currently lack integrative within-species studies that use quantitative proteomic methods, which allow rich multivariate descriptions of the SFP phenotype, to relate variation in SFP abundance to functional divergence, or SFP effectiveness [19]. A few previous studies have revealed differences in expression level of specific SFPs across populations [26, 32], suggesting that the precise compositon of the SFP cocktail may be a key male phenotype.

To improve the empirical foundation for an understanding of SFP evolution, we introduce a three-step empirical strategy to study within-species variation in SFP abundance that (1) contain the multivariate nature of the SF and (2) can be applied also to non-model taxa. First, quantitative proteomics methods [33] are employed to perform protein profiling of the SF-proteome of replicated SF samples from several populations/genotypes. Second, the resulting quantitative data on relative protein abundance is then analyzed using conventional multivariate statistical methods, to assess and characterize divergence in SFPs between types. Third, functional phenotypic in-vivo assays are used to associate differences in SFP abundance across types with differences across types in the reproductive efficacy of SF. This builds on a long-standing tradition in evolutionary biology to estimate natural selection by relating multivariate phenotypes to reproductive success.

Here, we illustrate the utility of this empirical strategy by employing it to study intraspecific variation in the SF-proteome of the seed beetle *Callosobruchus maculatus* (Coleoptera, Bruchidae). By profiling the male accessory reproductive gland proteome in replicated samples from 15 different populations, using quantitative 2D IEF SDS-PAGE analyses, we find that populations vary substantially in the male accessory gland proteome and that this variation is remarkably multidimensional. We then show that a large number of accessory gland proteins (i.e., Acps) are in fact transferred to females in the SF. Finally, we use functional assays to demonstrate that variation in SFP abundance across populations is closely associated with reproductive phenotypes, including measures of competitive male fertilization success and gonadotropic effects of SF in females. Our study provides important novel support for the sexual selection paradigm of SFP evolution.

## Results

### Proteomics assays

**Fig. 1:**
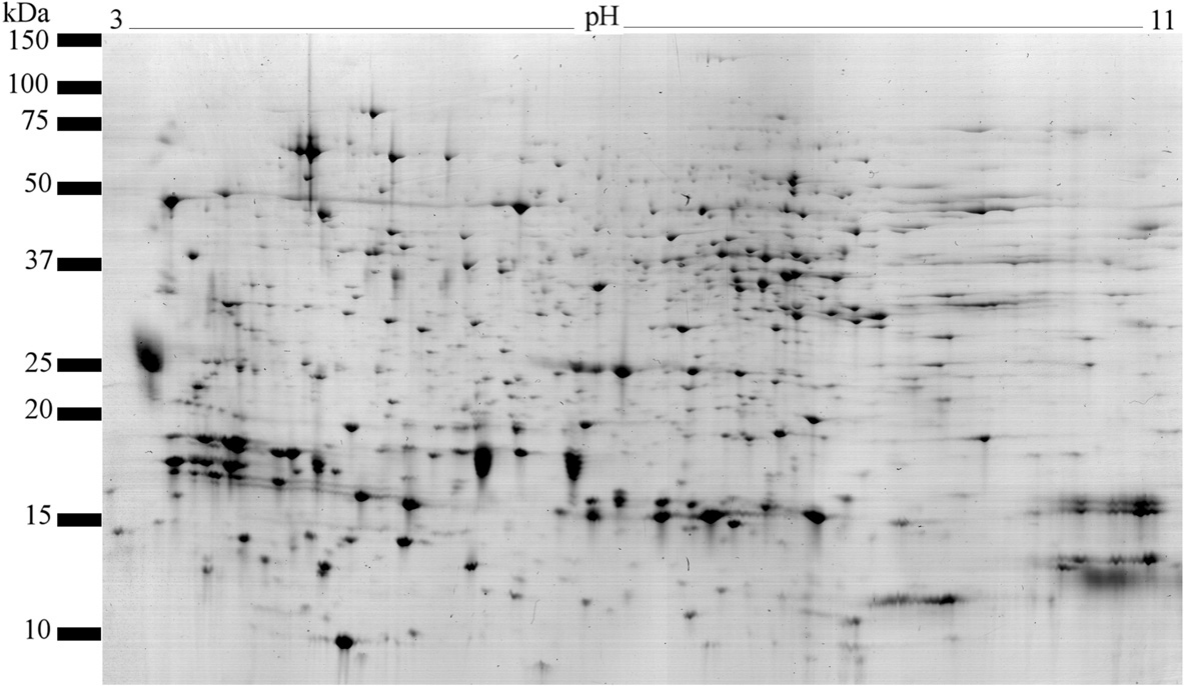
A representative gel image of the male accessory gland proteome of *C. maculatus*. The proteome was separated by 2D IEF SDS-PAGE and stained with Colloidal Coomassie blue

**Fig. 2:**
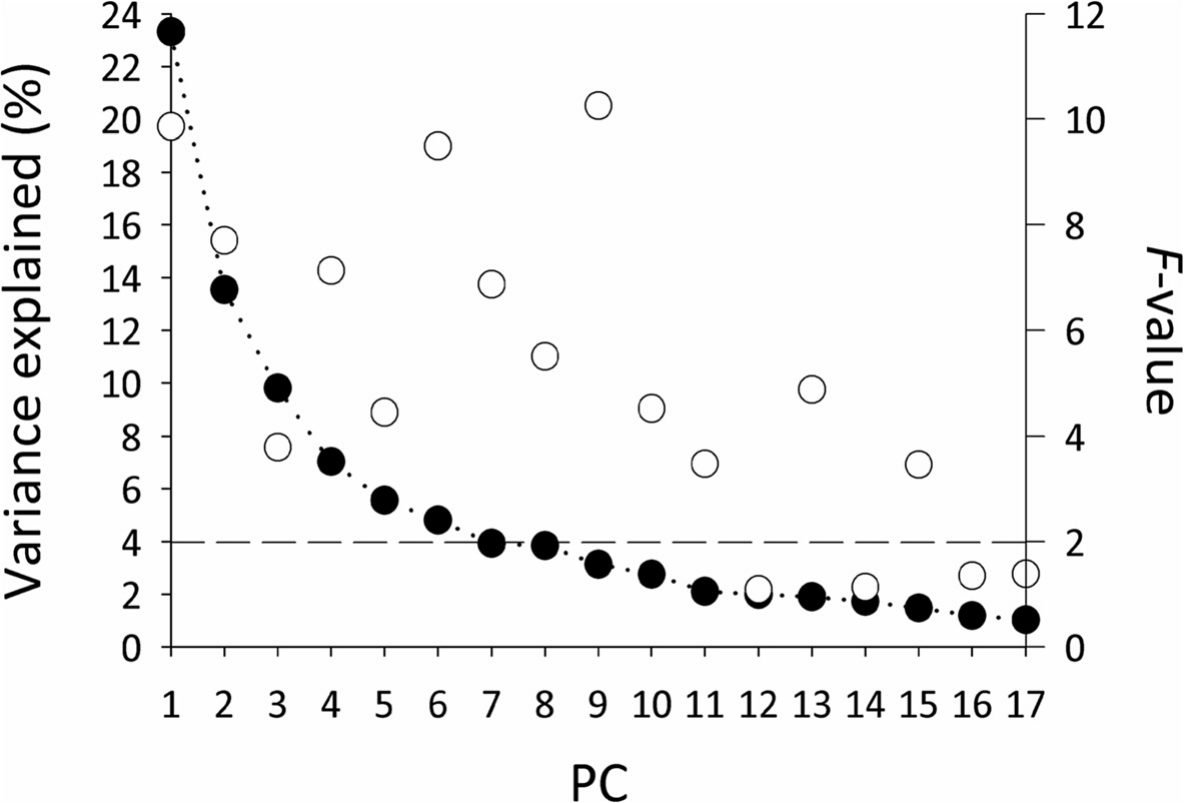
Scree plot from a principal component analysis of 683 male Acps. Given are the proportion of total variance in accessory gland proteins explained by successive PCs (filled circles) and *F*-values (open circles) from ANOVAs testing whether populations differ along each PC. Dashed line represents the critical *F* - value for *P* = 0.05

Our analysis showed that the male accessory gland proteome of *C. maculatus* is composed of at least 683 distinct Acps (Fig. 1). A PCA performed on the relative abundance of these 683 protein spots yielded 17 PCs, each of which accounted for >1 % of the total variance in normalized spot volume. Collectively, these 17 PCs accounted for more than 89 % of the total variance across all 60 replicate gels (Fig. 2). The repeatability of mean PC score per population was moderate to high (*r* = 0.46 − 0.74 and average *r* = 0.62 for PC 1 − 10), showing that a large proportion of variance in relative protein abundance across replicate gels was due to true variation between populations rather than to other sources of variation. Hence, our 2D IEF SDS-PAGE analyses of replicated gels showed sufficient accuracy. One-way ANOVAs of each of the 17 PCs revealed significant population differences in no less than 13 PCs (Fig. 2). These analyses reveal two major and novel insights. First, populations clearly differed markedly in their accessory gland proteome. Second, variation across populations in the accessory gland proteome was remarkably complex: the first 11 PCs differed significantly between populations. This unveils an extraordinary complexity. Because each PC captures a unique aspect of multivariate variation, it shows that significant differences across populations in the accessory gland proteome occur along many distinct multivariate dimensions. To better identify individual protein spots that differed across populations, we ran one-way ANOVAs for each of the 683 spots detected. After false discovery rate (FDR) compensation, significant differences in relative protein abundance occurred in 239 protein spots (q-value < 0.01). This shows that some 34 % of all Acps differed significantly in relative abundance across populations.

**Fig. 3:**
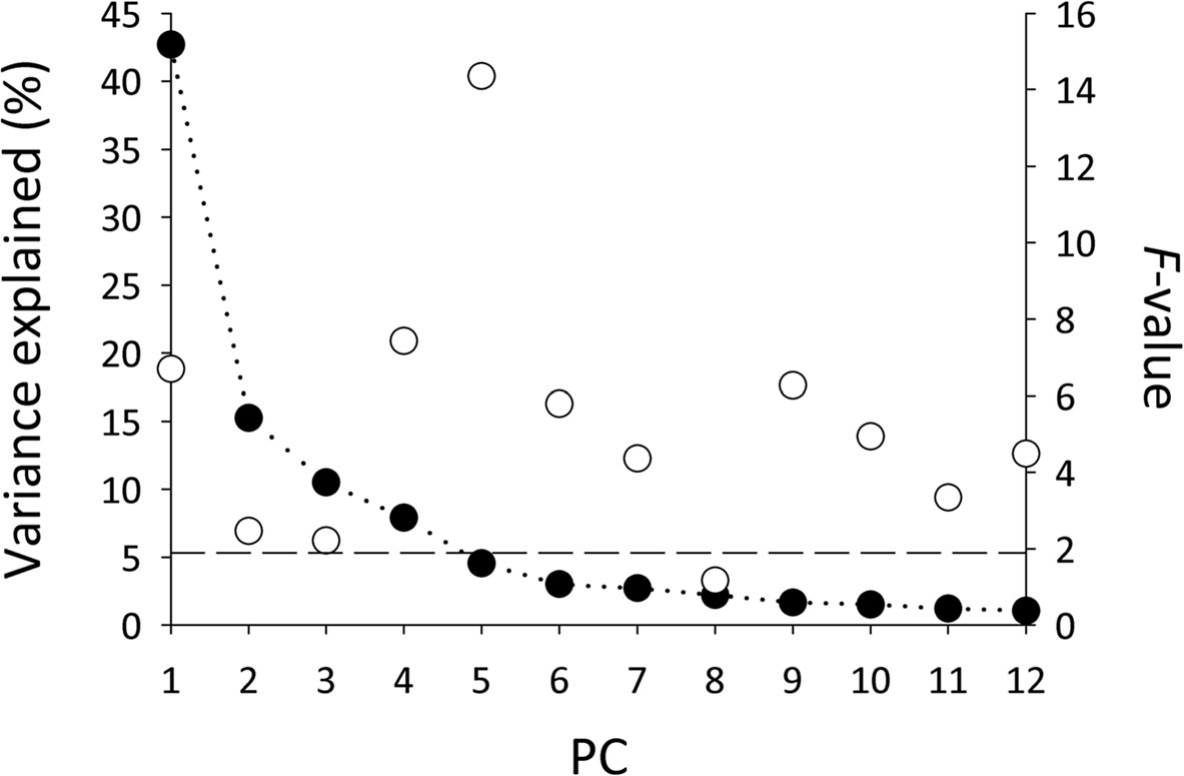
Scree plot from a principal component analysis of 127 seminal fluid proteins. Given are the proportion of total variance in SFPs explained by successive PCs (filled circles) and *F*-values (open circles) from ANOVAs testing whether populations differ along each PC. Dashed line represents the critical *F* - value for *P* = 0.05

The bursa copulatrix of just-mated females showed 508 unique and clear protein spots that did not at all occur in the bursa of virgin females. Some of these spots may represent proteins produced endogenously by females. However, because the time between the virgin state and the mated state was very short (<5 min), the large number of protein spots clearly shows that the ejaculate contains a highly diverse set of proteins. Preliminary proteomic analyses of the entire ejaculate (LC-MS/MS) suggest that it contains more than 400 proteins (unpublished observations). Out of the 508 spots detected here, 127 were identified as originating from male accessory glands. Thus, *C. maculatus* males transfer at least 127 SFPs to females during mating. Here, we refer to the 127 protein spots collectively as the seminal fluid (SF) proteome. A PCA performed on these 127 SFPs yielded 12 PCs, each of which accounted for >1 % of the total variance in normalized spot volume. Collectively, theses 12 PCs accounted for more than 94 % of the total variance in SFPs (Fig. 3). One-way ANOVAs of each of the 12 PCs revealed significant population differences in 11 PCs (Fig. 3). Again, these analyses unveiled striking and multifaceted differences across populations in the male SF proteome. One-way ANOVAs of each of the 127 SFPs showed that, following FDR compensation, the relative abundance of 56 of these proteins differed across populations (q-value < 0.05). Hence, almost half of all SFPs (44 %) differed in abundance across populations. Ongoing efforts to determine the molecular identity of these SFPs show that they belong to the biochemical classes of molecules commonly found in the seminal fluid of other taxa (e.g., metabolic enzymes, proteases, protease inhibitors, etc.) [4, 19].

Multivariate differences across populations in SFPs were not significantly related to differences between populations in either geographic distance, genetic distance or the number of years populations had been maintained in the laboratory (matrix correlations, │r│ < 0.3 in all cases; Mantel tests based on 10,000 iterations, *P* > 0.4 in all cases). Previous studies of these populations have also shown a lack of a phylogenetic signal in variation in reproductive phenotypes (e.g., [34]).

### Female fecundity

The population identity of males had significant effects on female fecundity, but this effect varied across time (Additional file 1: Table S1). To further characterize this pattern, we conducted separate analyses of female fecundity at three successive time periods (F^0^, F^1^ and F^2^; see Additional file 1: Table S2). These analyses showed that the male population effect was significant and strongest early in life, intermediate during mid-life and weakest late in life. Male genotypes thus differed in their ability to stimulate female egg production, primarily during the first 24 hrs following mating.

### Sperm competition success

Males from different populations differed significantly in their competitive sperm competition success, both in terms of sperm defense and offense (see Additional file 1: Table S3). This effect was relatively weak during the first 24 hours after the second mating, but was sizeable and significant after this initial period as well as for female lifetime egg production. Finally, the ejaculate weight of the second male was positively related to his competitive sperm competition success in the P_2_ assays (Additional file 1: Table S3).

### Association analyses

**Fig. 4:**
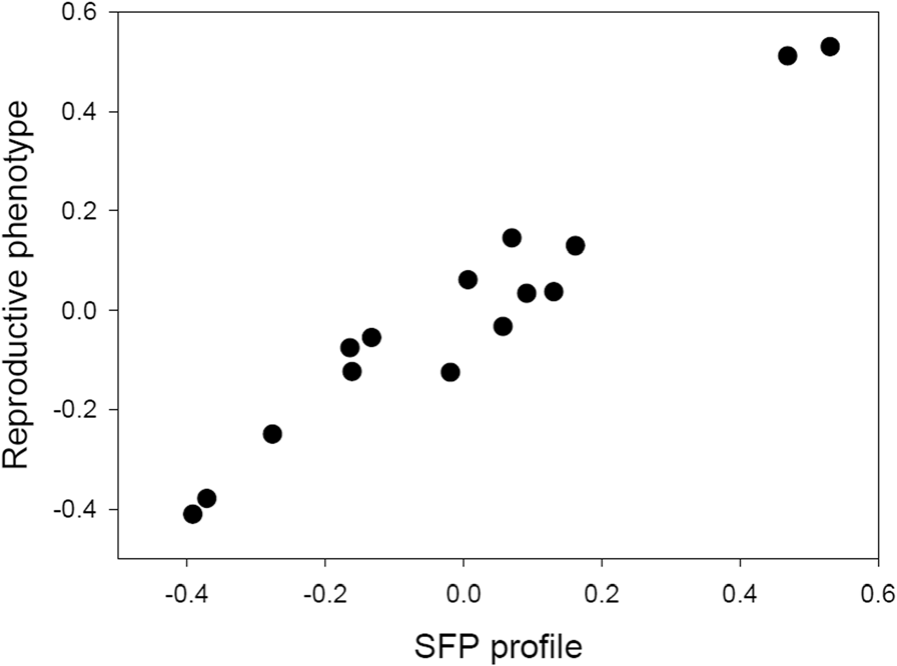
Multivariate relationship between male reproductive success and the seminal fluid composition. An ordination of the 15 *C. maculatus* populations studied here along the first pair of canonical variables, showing a significant covariation between the relative abundance of 56 SFPs (along the abscissa) and the reproductive phenotypic responses to seminal fluid (along the ordinate) (*R*_c_ = 0.97, Bartlett’s test: χ^2^_36_ = 56.7, *P* = 0.015). The second pair of canonical variables were marginally non-significant (*R*_c_ = 0.93, Bartlett’s test: χ^2^_25_ = 35.4, *P* = 0.081). A sizeable and significant proportion of total variance in reproductive phenotypes across populations was thus accounted for by variance in SFP abundance (Stewart-Love Canonical Redundancy Index: *Rd* = 0.713, bootstrapped 95 % CI: 0.61 − 0.80)

To assess whether variation in SFPs was functional, we conducted association analyses between variation in reproductive phenotypic variables and the relative abundance of SFPs across populations. Here, we used only the 56 SFPs and the six reproductive phenotypic variables that did in fact differ significantly across populations. We first performed an omnibus test of the overall hypothesis that SFPs and reproductive phenotypes covary, using a canonical correlation analysis. Because the dimensionality of the SFP matrix was too large relative to the number of populations studied, we first reduced the dimensionality of this matrix by means of a PCA based on the covariance matrix. This analysis showed that variation in SFPs (PC1-6; collectively explaining 91.5 % of the total variance) was indeed closely and significantly associated with reproductive phenotypes across populations (see Fig. 4). A closer inspection of univariate associations revealed many specific correlations. We found that 1 SFP was significantly associated with F^0^, 7 with F^1^, 5 with P_1_^1^, 6 with P_1_^T^, 6 with P_2_^1^ and 8 with P_2_^T^. There was some overlap, such that in total 20 out of 56 SFPs showed significant associations with at least one reproductive phenotype. Notably, a single SFP was correlated with measures of both P_1_ and P_2_ and two other SFPs were correlated with measures of both fecundity and P_2_. These analyses are summarized in Additional file 1: Figure S1. These associations, thus, demonstrate apparent functional redundancy across SFPs and show that some SFPs have pleiotropic reproductive effects.

## Discussion

Our study is the first to integrate an analysis of within-species divergence in the SF proteome with functional assays of reproductive phenotypes and our results provide a series of important insights. First, they unveil a high protein richness of the SF proteome. Second, the SF proteome differed markedly across populations and this difference was remarkably multidimensional. Third, we found that variation in the SF proteome was related to several important reproductive phenotypes. Fourth, our results provide a novel form of support for the sexual selection hypothesis of SFP evolution. Below, we discuss each of these points in some detail.

### The SF proteome

The male accessory gland is clearly a complex reproductive organ, producing many hundreds of proteins. Our analyses allowed us to distinguish the subset of Acps that are actually transferred to females at mating, which form an important component of the SF proteome. Using a conservative method, we show that at least 127 SFPs are transferred from the male accessory glands to females in *C. maculatus* and that the entire ejaculate likely contains more than 500 proteins. We note that high levels of protein richness in the seminal fluid have been reported in several other taxa, such as *Drosophila melanogaster* (>100 [1]), honeybees (>50, [35]), mosquitoes (>100, [36]), crickets (21, [37]) mice (69 [38]) and humans (923 [2]). Thus, it is clear that the SF proteome generally represents a very complex phenotype [19].

### Population divergence

Although rapid divergence between allopatric populations is a key prediction of several hypotheses for SFP evolution [11, 23, 24], we know of no previous large-scaled quantitative proteomic study of population divergence in the seminal fluid proteome. Baer et al. [26] found marked differences in abundance of several SFP across three populations of honeybees, but low replication did not permit a detailed quantitative analysis. Our study documented what must be considered to be very large differences across populations in the relative abundance of proteins among both Acps (Fig. 2) and SFPs (Fig. 3). Yet, we feel that the most striking aspect of population divergence seen here was its extraordinary complexity: significant population differences in the SF proteome occurred along multiple and orthogonal multivariate dimensions. In other words, populations were different from one another in many different ways. The fact that populations have diverged along many distinct multivariate trajectories has important bearing on hypotheses for the evolution of SFPs (see below).

### Function of SFPs

We show that overall variation in the SF proteome across populations was significantly related to important reproductive phenotypes, known to be affected by SFPs in this species [39] as well as in many other taxa [4]. Thus, our study provides important and novel support for the tenet that variation in SFPs is functional, in the sense that if affects reproductive success in both sexes. The main strength of association studies such as ours is that they are integrative and span the breadth even of complex phenotypes such as the SF proteome. However, it also suffers inferential constraints that derive from the fact that it is correlational. In contrast, experimental methods to ascribe function, for example using RNAi of single gene products or allelic variants of particular SFP genes, can provide stronger direct evidence for function of specific SFPs but suffer from an inability to handle complex and multidimensional phenotypes [19]. We note here that we have previously provided experimental evidence showing that the amount of SFPs that *C. maculatus* females receive affects both male competitive fertilization success (P_1_ and P_2_) and female egg production [39].

Variation across populations in sperm defense and offense abilities (i.e. P_1_ and P_2_) were related to several SFPs: six SFPs were associated with P_1_ and eight with P_2_, of which only a single SFP was associated with both. This suggests that the success in sperm defense and offense occurs through distinct mechanisms that act at different stages of fertilization. This is consistent with previous studies showing that different SFPs affect distinct reproductive events that may all influence male sperm competition success, such as sperm storage, retention and utilization as well as egg production and oviposition [19, 29–31, 40–44]. In *D. melanogaster*, for example, the sex-peptide (SP) modulates sperm release from the sperm storage organs and affects P1 [41], while Acps36DE facilitates sperm storage thus affecting both P_1_ and P_2_ [42, 44]. Moreover, Acps33A, Acp29AB, CG17331, CG6168, CG14560 and Acp62F have been shown to affect P_1_ and/or P_2_ through their effects on female sperm handling [30, 31, 45, 46]. A recent study of *C. maculatus* showed that two different size-fractions of accessory gland proteins affected P_2_ [39]. The fact that several different SFPs were associated with P_1_ and P_2_ in the current study provides evidence for functional redundancy [19, 31, 47], defined as functional duplication [19], in the sense that several SFPs affected the same reproductive phenotype. We note, however, that the extent to which this represents lower-level redundancy is unclear, as reproductive phenotypes such as P_1_ may in itself result from a multitude of underlying physiological processes.

We also observed apparent functional redundancy for associations with fecundity. For example, no less than seven SFPs were associated with female fecundity the second day after mating while a single SFP was associated with fecundity the first day. Again, this is consistent with the fact that female egg production requires oogenesis, ovulation and oviposition [4, 19, 48]. In *D. melanogaster*, ovulin (Acp26Aa) and SP both stimulate egg production. Ovulin induces the release of mature oocytes from the ovary within the first 24 hrs following mating, whereas SP stimulates egg production for several days after mating [49–52]. In line with our findings, Yamane et al. [39] recently demonstrated effects of different size fractions of accessory gland extract on egg deposition during the first and second day, respectively, in *C. maculatus*. Finally, we note that the apparent functional redundancy detected here did not seem to reflect concerted up-regulation of any single pathway, since a cluster analysis of variation in protein abundance across gels showed that the SFPs that were associated with a given phenotype did not generally tend to cluster together (Additional file 1: Figure S2).

We also identified three SFPs that apparently showed pleiotropic effects (31). One SFP had congruent effects on both P_1_ and P_2_ and, intriguingly, two SFPs had antagonistic effects on fecundity and P_2_. Interestingly, Yamane et al. [39] found that some size-fractions of SFPs both stimulated egg laying and reduced P_2_ in *C. maculatus*. They suggested that this antagonistic effect may occur because oviposition can interfere with sperm uptake to the spermatheca as eggs pass down the oviduct.

### Sexual selection and the SF proteome

The postmating sexual selection paradigm for the evolution of SFPs [6, 17, 18, 29] relies on phenotypic selection on SFPs, such that natural within-species variation in the multivariate SFPs phenotype (i.e., the type and amount of SPFs transferred) is related to male and female postmating reproductive success. To our knowledge, our study provides the first integrative evidence for this critical component of the paradigm. In this important regard, our findings are very difficult to reconcile with the alternative hypotheses that (1) host-pathogen coevolution is driving evolution SFPs [6, 53] or that (2) the evolution SFPs is an incidental side-effect of relaxed selection due to sex-limited gene expression [54].

The sexual selection paradigm, then, is based on the fact that evolutionary modifications of the seminal fluid that provides postmating benefits to males will be favored by male-specific selection [55]. This may involve modifications of the expression levels and/or changes in form/structure of SFPs [19]. Because male and female postmating interests often diverge [17, 19], some of these modifications will have sexually antagonistic effects. Incidentally, SFPs with toxic side effects in females have been documented in other seed beetle species [56, 57]. The resulting sexual conflict will then generate selection in females to alleviate any detrimental effects of SF substances, sparking rapid and diversifying sexually antagonistic coevolution [17, 19]. As recently stressed by Sirot et al. [19], observations of complexity and redundancy of the SF proteome are consistent with this scenario. Because SFPs serve as agents that manipulate many different postmating aspects of female physiology and behavior, and because any novel SFP that benefits males will be favored by selection, we predict the evolution of a very diverse and at least partly redundant set of SFPs. With such a multivariate phenotype, random mutation-order events [28] may then lead male-female coevolution along different multivariate trajectories in different populations which may significantly contribute to the evolution of reproductive isolation [15, 16, 58]. Two additional and novel aspects of the intraspecific divergence seen in our study are noteworthy in this context. First, the evolution of SFP expression appears rapid in *C. maculatus*. In fact, the effect size of divergence across these populations in the SF proteome is much larger than those seen previously for other phenotypes such as morphology [59, 60] and life-history traits [34, 61]. Second, population divergence in SFP expression is remarkably multidimensional. The fact that the SF proteome, as well as the Acp proteome, differs between populations along so many different (orthogonal) dimensions shows that evolution occurs along a multitude of multivariate trajectories. Previous observations of con-specific and con-population sperm precedence in the genus *Callosobruchus*, which has been presumed to be mediated through seminal fluid [62–64], suggest that the processes that generate the population divergence in SFPs seen here will also contribute to speciation in this group.

Those female molecules that interact with SFPs (i.e., receptors) play a crucial role in the postmating sexual selection paradigm. This is because without evolutionary modification of female traits, there would be no male-female coevolution [65] and thus little evolutionary diversification [66]. Unfortunately, we currently know little about the evolution of female receptors for SFPs [19], simply because very few have been identified [67, 68]. In our functional assays, we used a standard reference female genotype with a given reproductive response repertoire to the different SF proteomes. However, studies of crosses between genotypes in *C. maculatus* have shown that females differ in their postmating response to a given male genotype [68–70]. This strongly suggests that female receptors also differ across populations, potentially as much as do male SFPs. Revealing the full coevolutionary complexity of male-female interactions will prove a major challenge, as such studies will need to quantify not only the multidimensional male SFP phenotype dealt with here but also the multidimensional female receptor phenotype.

## Conclusions

An understanding of the divergent evolution of SFPs requires knowledge of the mechanism of selection that acts upon variation in this class of important reproductive proteins. Sirot et al. [19] recently suggested that within-species studies of variation in the SF proteome and associated reproductive responses can advance this field. Here, we delineate an empirical strategy whereby such advances can be gained and we provide an application of this framework in a seed beetle. We show that within-species variation in the SF proteome was associated with the efficacy of important reproductive effects of the SF, thus providing the first direct evidence for postmating sexual selection on relative SFP abundance. Differences in the SF proteome across populations were large and multifaceted, consistent with the rapid and multidimensional evolution of SFPs predicted by the postmating sexual selection paradigm of SFP evolution. Our results illustrate the importance of intraspecific studies of SFPs and we suggest that similar studies performed in other systems would significantly help expose the processes by which SFPs evolve.

## Methods

### Stocks

We used 15 different focal populations of *C. maculatus*, with distinct geographic origin (Brazil I, Brazil II, California, IITA Nigeria, Lomé, Mali, Nigeria Mix, Ofuya, Oman, Oyo, South India, Uganda, Upper Volta, Yemen, Zaire), as well as a standard reference stock (hence, SRS) population (SI USA). Populations were provided by Peter Credland (University of London, UK), Robert Smith, (University of Leicester, UK), Thomas Ofuya (Federal University of Technology, Nigeria) and Glitho Adolé (Université de Lomé, Togo). These populations are closely related [61] and reproductively fully compatible: they are phenotypically very similar and crosses show normal egg-adult viability (>90 %) [63, 70]. Geographical distances between populations were calculated using the Geographic Distance Matrix Generator [71]. Focal populations were reared under common garden conditions on black-eyed beans (*Vigna unguiculata*), the SRS on mung beans (*Vigna radiate*) and all beetles were reared in the laboratory at 29 °C, 60 % RH and a 12 L:12D light cycle. Assays were done under the same conditions. Ethics approval is not required for the research we report here, as it involves an insect species for which no ethical restrictions apply.

### Male accessory gland proteome

To examine whether and how the SF-proteome differs across the 15 focal populations, we used a proteomic approach implementing two-dimensional polyacrylamide gel electrophoresis (2D IEF SDS-PAGE). Here, proteins are effectively separated along two orthogonal gradients (mass and pH) and subsequent staining and image analysis allows measures of protein spots on gels. Although this method is relatively crude and less informative than some alternatives (e.g. LC-MS/MS), it allows quantitative analyses of variation in protein abundance phenotypes also in non-model taxa where well annotated genomes are not present. A critical component of our study was replication, which allowed validation of our measures of relative protein abundance. We note that, in theory, spot variation between 2D IEF SDS-PAGE gels results primarily from quantitative differences in protein abundance, but may also be affected by e.g. differential post-translational modification, protein sequence differences or differential splicing. This is less of concern here, however, as we focus on variation in the integrated SFP phenotype rather than on precisely what such differences represent.

Given that SFPs are primarily produced in male accessory reproductive glands in arthropods in general [51] and in seed beetles in particular [72], we used these glands to characterize the male reproductive proteome. We first assessed divergence in the entire accessory gland proteome and then focused on the subset of Acps that were in fact transferred to females.

### Sample preparation

Accessory reproductive glands of virgin males (0-1 day old), held in isolation, were dissected out in insect saline on ice, kept in Eppendorf tube with 60 μl of sterile Milli-Q water on ice for the duration of the dissecting period (not more than 20 minutes), and were then frozen at -80 °C prior to protein extraction. For each biological replicate, we pooled 60 pairs of accessory glands originating from 60 individual males. The frozen glands were thawed and mechanically homogenized using a pestle in 120 μl of buffer (8 M urea, 4 % CHAPS and 0.002 % bromophenol blue (BFB)). Samples were then centrifuged at 13,000 rpm for 7 min at 4 °C, following the method implemented by Takemori and Yamamoto [73]. The supernatant containing the soluble proteins was then subjected to 2D electrophoresis. In total, we analyzed four independent biological replicates per population to allow downstream statistical analyses of the accuracy of our multivariate protein abundance measures and of variation across populations.

### 2D gel electrophoresis

To each replicate sample, we first added buffer to a final sample volume of 450 μl. We then added IPG and dithiothreitol to achieve a final concentration of 0.5 % and 20 mM, respectively. The samples were then incubated at room temperature (for 30 min). Immediately prior to starting the 1^st^ gel dimension, each sample was again centrifuged at 12,500 rpm for 5 min. The supernatant was then loaded on a pH3-pH11 NL 24-cm strip (Immobiline DryStrips, GE Healthcare). The samples were run to a total of 64 000 Volt-hrs, which took 10.5 hrs. Following isoelectric focusing (IEF), IEF strips were incubated during 15 min in equilibration solution (100 mg dithiothreitol in 10 ml: 6 M Urea, 75 mM Tris-HCl pH 8.8, 2 % (w/v) SDS, 30 % (v/v) Glycerol, 0.002 % BFB) and for an additional 15 min in a second solution (250 mg Iodoacetamine in 10 ml: 6 M Urea, 75 mM Tris pH 8.8, 2 % (w/v) SDS, 30 % (v/v) Glycerol, 0.002 % BFB). The IEF strips were then transferred onto a polyacrylamide gel, and the the 2^nd^ dimension was run using ExcelGel XL SDS 12-14 % (GE Healthcare) and buffer strips (ExcelGel XL, GE Healthcare). The gels were run horizontally at 200 V, 20 mA and 20 W per gel for 40 min and then at 800 V, 40 mA and 40 W for the following 2 hrs 40 min. Each gel was then incubated in fixing solution (10 % methanol, 7 % acetic acid) for 30 min, rinsed with water for 30 min several times after fixation, and stained overnight with Colloidal Coomassie Blue.

### Image analysis

All 60 stained gels (15 populations × 4 biological replicates) were scanned with ImageScanner (GE Healthcare) at 300 dpi and the gel images were subsequently analyzed using Progenesis SameSpots software (Nonlinear Dynamics, Newcastle, UK). A single aberrant gel image was deemed oversaturated and was excluded from further analysis. All gel images were aligned against a reference gel, using a semi-automated procedure in which 30 landmarks were manually added to guide the automated alignment. Protein spot detection on the gel images was performed using default setting for detection, background subtraction, normalization and matching. Spots detected in all, or all but one, gels from a given population were considered valid protein spots. Each protein spot was carefully inspected in detail, based on peak height and 3D visualization, and then manually edited to exclude artifacts if needed (i.e., splitting of closely located but distinct spots or merging of single true spots). Normalization was restored following editing. The normalized volume of each protein spot from each of the gel images was used as a measure of relative protein abundance in subsequent statistical analyses.

### Data analysis

We used standard one-way ANOVAs to identify those Acps whose relative abundance differed most among populations, using the normalized protein spot volumes as the response variable. Resulting inferential tests were FDR compensated for multiple comparisons using Storey’s method as implemented in the Qvalue package in R [74].

To characterize multivariate variation in the accessory gland proteome, we reduced the dimensionality of the protein spot volume matrix by a principal component analysis (PCA), based on the on the covariance matrix and using JMP version 10.0 (SAS Institute Inc., Cary, NC, U.S.A.). This analysis generates latent variables (i.e., PCs) that are orthogonal and, thus, capture unique and distinct aspects of multivariate variation in the accessory gland proteome. To test whether the accessory gland proteome differed among populations, we performed one-way ANOVAs of these PCs.

### Female reproductive tract proteome

To identify the subset of Acps that are actually transferred to females during mating, and are thus SFPs, we analyzed the protein profile of the female reproductive tract in mated and virgin females using a 2D IEF SDS-PAGE approach and compared this to the male accessory gland proteome. Because the male ejaculate in *C. maculatus* is very large, constituting approximately 5 % of the adult male body weight [75], it contributes with a sizeable portion of the total amount of proteins contained within the reproductive tract of just-mated females.

### Sample preparation

Virgin SRS females (0-1 day-old) were individually mated with virgin males (0-1 day-old) from a focal population. Immediately after mating, which lasts for 3-5 minutes in this species, each female was placed in an Eppendorf tube and stored on ice until we collected 5 females that were mated with males originating from a given focal populations (<5 min on ice). Females were then frozen at -20 °C. Virgin SRS females were simultaneously frozen at -20 °C. The bursa copulatrix (the organ which receives the ejaculate) of virgin and mated females were dissected out in insect saline on ice, rinsed and stored in a tube with 60 μl sterile Milli-Q water on ice during the dissecting period (< 20 min). Samples were then immediately frozen at −80 °C until protein extraction. For each biological replicate, we pooled either 75 bursae copulatrices from virgin or 75 bursa copulatrices from mated females (i.e., 15 populations × 5 mates of males from each population). The frozen bursas were thawed and protein extraction and 2D gel electrophoresis was performed as described above for male accessory glands. In total two independent biological replicates for mated and two for virgin females were analyzed.

### Image analysis

Here, we first generated the spot pattern in mated females, then the spot pattern in virgin females and finally removed all spots occurring in virgin females from those in mated females. The “difference” between these two sets of gels thus represents candidate male-derived SFPs. In the final step, we superimposed the spot pattern of these candidate SFPs in gels of mated females on the spot pattern in gels of male accessory glands. Those protein spots that occurred both in the bursa copulatrix of mated females and in male accessory glands, but were lacking in the bursa copulatrix of virgin females, were here deemed SFPs.

The four stained female gels were scanned with ImageScanner (GE Healthcare, Uppsala) at 300 dpi and the analysis of gel images was performed using Progenesis SameSpots software (Nonlinear Dynamics). First, one of the gels from mated female was selected as a reference gel and the remaining gel images were aligned with the reference gel using a semi-automated procedure in which 20 landmarks were added manually to guide the alignment. Second, we obtained the spot pattern for gels of mated females and then stamped this pattern over gels of virgin females. Spot detection was performed using default setting for detection, background subtraction, normalization and matching. Third, we manually removed all spots that occurred in both virgin and mated females, thus obtaining the candidate SFP spot pattern. Fourth, we manually aligned the gel image of mated females with that of the reference gel of male accessory glands. Finally, the candidate SFP spot pattern was overlapped with the male accessory gland spot pattern (see above). The overlapping spots between these two sets of spot patterns represents SFPs transferred to females at mating. We note that this inferential strategy, in 1D, has previously been used to successfully identify SFPs in *Aedes aegypti* and *Tribolium castaneum* [76, 77].

We stress that the method used here to identify SFPs is conservative, in the sense that it underestimates the total number of proteins present in the seminal fluid, for five reasons. First, we excluded a few candidate SFPs where the spot volume was larger in females than in males. Second, a few candidate SFPs that showed a very weak spot in females were excluded. Third, some SFPs are produced also in testes and/or in the seminal vesicle. These are, obviously, not included in our set of SFPs. Fourth, posttranslational but premating modification of some SFPs may occur in males, for example in the seminal vesicles. Fifth, some protein spots detected in our 2D IEF SDS-PAGE analyses are known to contain more than a single distinct protein.

### Data analysis

We tested for a difference between populations in the subsection of the male accessory gland proteome that was deemed to constitute SFPs. First, we used standard one-way ANOVAs of normalized spot volumes to test for a difference in SFPs across populations. Resulting inferential tests were FDR compensated for multiple comparisons using Storey’s method as implemented in the Qvalue package in R [74]. Second, we reduced the dimensionality of the SFP spot volume matrix by a principal component analysis (PCA) (as above). To test whether SFPs differ among populations, we performed one-way ANOVAs of the resulting PCs.

We also calculated the SFP proteomic distance matrix between populations, as the multivariate Euclidean distance between normalized spot volumes. We then used matrix correlation analyses to relate this distance with geographic distance (see above), genetic distance (determined from a 1008 bp fragment of COI [78]) and differences in the number of years populations had been maintained in the laboratory. These matrix correlations test whether populations that differ more in SFPs are also more different in other regards.

### Phenotypic assays

We assayed two sets of reproductive phenotypes, male ability to simulate female fecundity and male sperm competition success, both known to be affected by SFPs in *C. maculatus* [39]. Because we focus on SFP variation across focal males, the reproductive phenotypes were assayed in a common standard female background (i.e., our SRS). The generation prior to each of our phenotypic assays, 200-300 adults of each population were transferred into a stock jar provided with beans and were allowed to oviposit for 24 hr. We then isolated single beans (96 from each focal population and 900 from the SRS population) carrying eggs in 48-well tissue culture plates. After 23 days, virgin males and females were collected once every day from these plates and were later used in the phenotypic assays detailed below.

### Female fecundity

Male ability to stimulate egg laying in SRS females was estimated in a series of mating trials. Here, we measured female offspring production in early-, mid- and late-life. For each focal population, we placed a virgin male (total N = 36-40 per focal population) with a virgin SRS female (body weight measured to the nearest 0.00001 g, using a Sartorius® Genius ME 235P microbalance) in a Petri dish until they mated. Once mating was terminated, the female was transferred to a Petri dish with 50 beans and kept there for 24 hrs (fecundity at 0 - 24 hrs, F^0^). Following this period, the female was transferred to a dish with 50 new beans for the following 24 hrs (fecundity at 24 - 48 hrs, F^1^). Finally, the female was mated a second time with a virgin male (0 - 1 days old) originating from the same focal population as her first mate. The female was then placed in a dish containing 100 beans and were kept there until death (fecundity at 48 hrs - death, F^2^). Female lifespan was recorded daily. All hatching offspring were recorded in each of the three dishes collected from each female and were used as measures of female fecundity. Because distinct SFPs stimulate female egg production early and late in life in *C. maculatus* [39], the fact that we measured offspring production at different female ages allowed us to identify distinct candidate SFPs for early and late life female fecundity stimulation.

### Sperm competition success

To gain population-specific estimates of male competitive fertilization success, we measured both defense (P_1_) and offense (P_2_) components of sperm competition success against a standard sperm competitive background. We used a standard sterile male technique [79] in which SRS females are mated to two males in succession, one of which is sterilized and one of which is not. Here, P_1_ and P_2_ refer to experiments where the focal male is the first or the second male to mate, respectively, with a given female in such double mating experiment. Because distinct SFPs affect male fertilization success in females of different age [39], we estimated both short- and long-term effects. To estimate P_1_, virgin SRS females (0-1 day old) were each mated once to a focal virgin male (0-1 day old) from a focal population. Immediately after mating, females were isolated with 50 beans for 24 hrs, after which each female was remated a second time with a sterile SRS male (0-1 day old; sterilized by exposures to gamma radiation using a caesium source; dose 100 Grey, known to cause complete sterility without impairing sperm motility/viability [80]) and then placed in a Petri dish containing 50 beans for the next 24 hr. After this time, females were transferred to a new dish containing 100 beans where they were kept until death. Life span was recorded daily. In total, we conducted 24-30 replicates per population. We then estimated P_2_ in a series of separate assays using the same exact protocol, with the exception that sterile SRS males were first to mate and focal virgin males were second to mate. Here, we conducted 6-11 replicates for each focal population. For both P_1_ and P_2_, we estimated competitive fertilization success separately based on eggs laid 0 - 24 hrs (P^0^) and 24 hrs onwards (P^1^) after the second mating, as well as the sum of the two (P^T^).

For both P_1_ and P_2_ assays, we subsequently recorded egg hatching. All populations used here normally show egg hatching rates >97 %. Hatched and unhatched eggs were thus considered to have been fertilized by the fertile focal and sterile SRS males, respectively. The proportion of hatched eggs thus provides a direct measure of P_1_ and P_2_ of the focal male. The number of eggs laid between the first and the second mating was recorded and used as a covariate in our models, as this is known to affect competitive fertilization success in *C. maculatus* [81]. Moreover, female body weight as well as the ejaculate weight of both males were recorded and used as covariates in the inferential models described below.

### Data analysis

A covariation between SFPs and reproductive phenotypes across populations relies on populations differing significantly in both. We thus first asked whether the reproductive phenotypes measured differ across populations. The population effect of mating on female fecundity was analyzed in a univariate repeated measures ANOVA, in which population and block were between-subjects factors and time was a within-subject factor. Life span and female body weight were both included as continuous covariates in this model. We used Greenhouse-Geisser adjusted *P*-values for all within-subjects effects. To further characterize effects, we conducted separate two-way ANOVAs for each response variable (F^0^, F^1^ and F^2^) with population and block as main factors and life span and female body weight as covariates. A few deviant females, with standardized residuals │R│ > 3.0, were excluded from these models.

We tested for a difference across populations in P_1_ and P_2_ at each time-point using generalized linear models, with binomial errors and logit link functions. The number of hatched eggs was used as the nominator and the total number of eggs laid as the binominal denominator. To compensate for overdispersion, we used a Pearson χ^2^ adjustment prior to statistical inference. These models included population and block (when appropriate) as factorial variables as well as the four covariates described above. A few females, that either laid no eggs between the first and second mating or that laid no hatching eggs, were excluded from these models.

### Association analyses

A key element of our analytical strategy is associating variation in SFPs with variation in reproductive phenotypes that might be affected by SFPs across populations. To achieve this, we related population specific mean values of those reproductive phenotypes that differed significantly across populations with the population specific mean normalized spot volumes of those SFPs whose relative abundance differed significantly across populations. We term those SFPs that showed a significant association with reproductive phenotypes functional SFPs, given variation in their relative abundance correlated with reproductive phenotypes.

### Availability of supporting data

All supporting data for this article are included in the additional file.

## Additional file

Additional file 1:
**Supplementary supporting information for Goenaga et al.** (Figure S1: Associations between SFPs and reproductive phenotypes; Figure S2: Hierarchical clustering of SFPs abundance across gels; Tables S1-S3: Statistical analysis of variation in reproductive phenotypes across populations). (PDF 344 kb)
