# Supplementary material for: Within-species divergence in the seminal fluid proteome and its effect on male and female reproduction in a beetle
Source: BMC Evol Biol. 2015 Dec 2;15:266. doi: 10.1186/s12862-015-0547-2 (PMC4667481; doi:10.1186/s12862-015-0547-2)
Supplement: Additional file 1: — Supplementary supporting information for Goenaga et al. (Figure S1: Associations between SFPs and reproductive phenotypes; Figure S2: Hierarchical clustering of SFPs abundance across gels; Tables S1-S3: Statistical analysis of variation in reproductive phenotypes across populations). (PDF 344 kb) [file 12862_2015_547_MOESM1_ESM.pdf]

Figure S1. Associations between those 56 SFPs and reproductive phenotypes that showed significant differences across populations, measured as pairwise correlations. Given are F^0 (A), F^1 (B), P_1^1(C), P_1^T(D), P_2^1 (E) and P_2^T (F) (see main text for description of variables). Solid lines represent the critical *r* - value for *P* = 0.05. Note that SFP number is arbitrary, although consistent across plots.

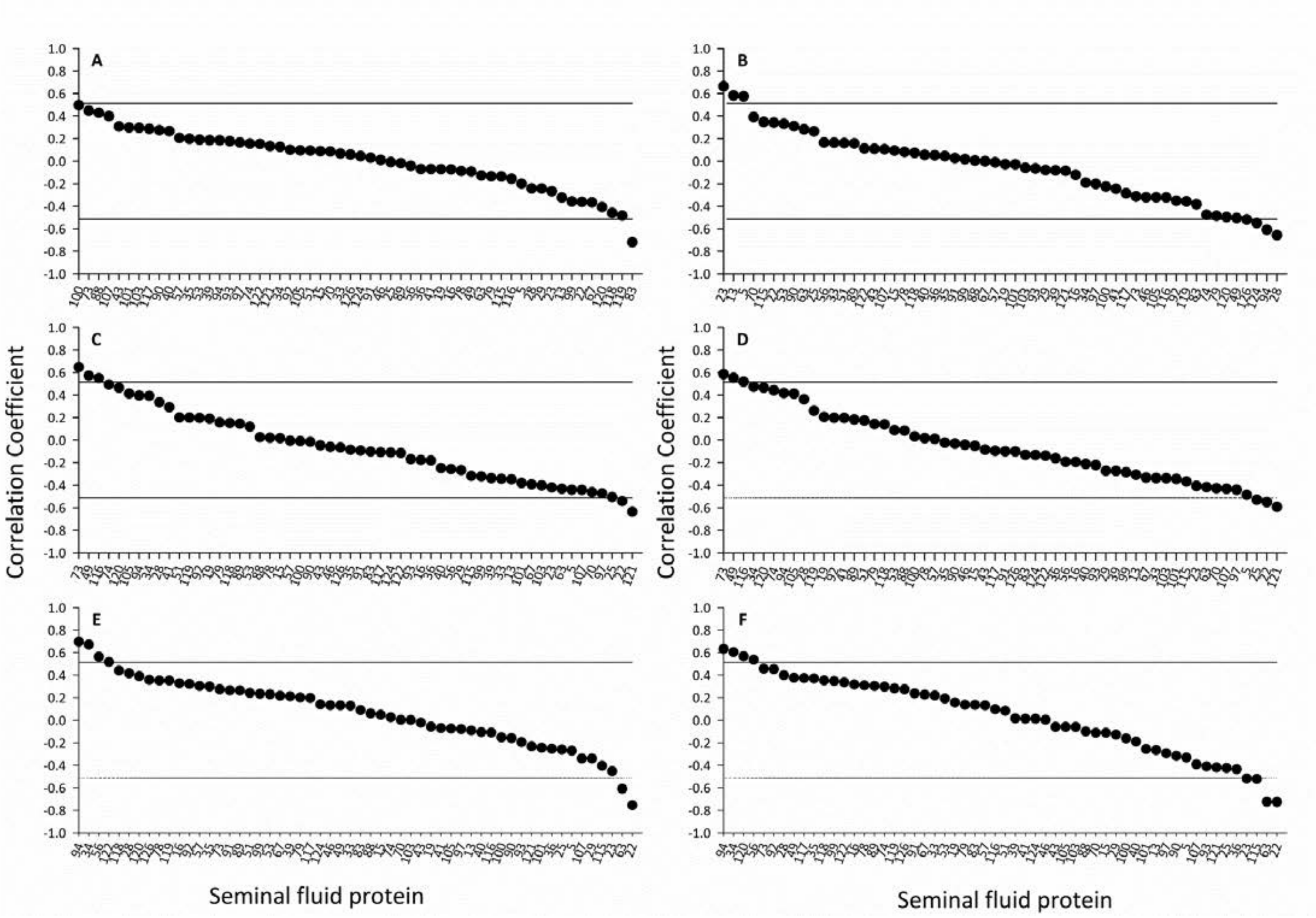

Figure S2. Hierarchical clustering of SFPs abundance across gels, based on the combined Euclidean distance and Ward's method. Colors indicate those SFPs that were significantly associated with reproductive phenotypes. Given are F^0 (pink), F^1 (red), P_1^1 (blue), P_1^T (light blue), P_2^1 (green) and P_2^T (light green) (see main text for description of variables).

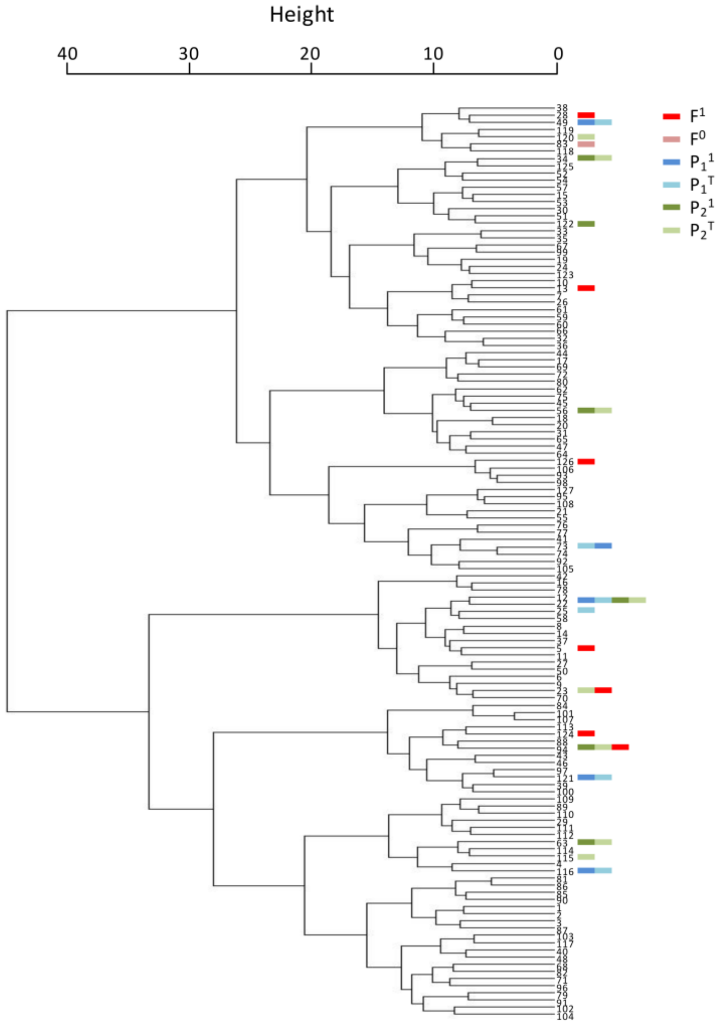

Table S1. Repeated measures ANOVA of female fecundity at three different time-periods. Greenhouse-Geisser adjusted *P*-values within brackets.

Source	df	*F*	*P*
Between subjects			
Population	14	0.893	0.5650
Block	1	58.0251	<0.001
Population × Block	14	1.311	0.195
Lifespan	1	45.609	<0.001
Female body weight	1	36.058	<0.001
Error	521		
Within subjects			
Time	2	348.005	<0.001 (<0.001)
Time × Population	28	1.820	0.005 (0.012)
Time × Block	2	16.861	<0.001 (<0.001)
Time × Population × Block	28	1.291	0.1427 (0.168)
Time × Lifespan	2	17.964	<0.001 (<0.001)
Time × Female body weight	2	22.201	<0.001 (<0.001)
Error	1042		

Table S2. Two-way ANOVAs of female fecundity during each of three successive time-periods of their life.

	F^0			F^1			F^2		
Sources	df	*F*	*P*	df	*F*	*P*	df	*F*	*P*
Population	14	2.255	0.005	14	1.591	0.0773	14	1.366	0.164
Block	1	2.476	0.116	1	23.176	<0.001	1	38.927	<0.001
Population × Block	14	0.854	0.609	14	1.514	0.101	14	1.252	0.233
Lifespan	1	0.001	0.981	1	1.221	0.265	1	48.829	<0.001
Female body weight	1	0.709	0.400	1	0.848	0.354	1	50.614	<0.001
Error	527			527			525		

Table S3. Generalized linear models of the effect of population on P_1 and P_2, given separately for two time-periods as well as for the total life span of females. Ejaculate weights refer to standard reference (SRS) and focal (F) males.

	P_1^0			P_1^1			P_1^T		
Source	df	Deviance ratio	*P*	df	Deviance ratio	*P*	df	Deviance ratio	*P*
Population	14	1.35	0.174	14	2.03	0.015	14	1.85	0.030
Block	1	0.08	0.784	1	7.94	0.005	1	4.19	0.041
Block x Population	14	1.77	0.042	14	1.72	0.051	14	1.81	0.036
No eggs between 1st and 2nd mating	1	2.76	0.098	1	0.91	0.341	1	2.08	0.123
Ejaculate weight ♂SRS	1	0.01	0.920	1	0.10	0.747	1	0.06	0.808
Ejaculate weight ♂F	1	0.99	0.321	1	3.40	0.066	1	2.24	0.135
Female body weight	1	2.20	0.139	1	2.35	0.126	1	2.40	0.122
Residual	369			366			369		

	P_2^0			P_2^1			P_2^T		
Source	df	Deviance ratio	*P*	df	Deviance ratio	*P*	df	Deviance ratio	*P*
Population	14	1.36	0.191	14	6.86	<0.001	14	2.97	<0.001
Ejaculate weight ♂F	1	6.31	0.014	1	17.5	<0.001	1	10.70	0.002
Ejaculate weight ♂SRS	1	1.03	0.314	1	3.9	0.050			
No eggs between 1st and 2nd mating	1	4.82	0.031	1	3.76	0.056	1	4.86	0.030
Residual	82			84			88		
